# Nursing in Columbia

**Published:** 1878-02

**Authors:** 


### NURSING IN COLUMBIA.

Dr. André Posada-Araryo sends to the president of the society for the protection of infancy in Paris, a letter from which we extract the following passages: "There is neither law nor society for the protection of infants in Columbia. The profession of wet-nurses does not exist here. Every woman, rich and poor is accustomed to suckle her child until the signs of a new pregnancy appear, which happens ordinarily at the ninth month, so that each child is eighteen months older than its successor. There are a great many women giving birth, every eleven months, to children who do well. Nursing does not interfere at all with procreation. Each marriage here (state of Antioqua, Columbia) produces 10, 12, or 15 children. There is one woman, who has had 34 children, and they are all living (she had several times twins). Her descendants as far as the great grand-sons comprise an immense number. I also know a man who has been married three times, and can count already 51 children. His wife is still young and he may be able yet to reach 60. The women here marry early—from 13 to 16 years of age. They commence to menstruate at the 13th or 14th year.

